# A meta-analysis comparing cisplatin-based to carboplatin-based chemotherapy in moderate to advanced squamous cell carcinoma of head and neck (SCCHN)

**DOI:** 10.18632/oncotarget.6858

**Published:** 2016-01-09

**Authors:** Jian Guan, Qinyang Li, Yue Zhang, Nanjie Xiao, Min Chen, Yaowei Zhang, Lu Li, Longhua Chen

**Affiliations:** ^1^ Department of Radiation Oncology, Nanfang Hospital, Southern Medical University, Guangzhou, China

**Keywords:** carboplatin, cisplatin, meta-analysis, head and neck, cancer

## Abstract

**Purpose:**

This study was performed to compare the efficacies and toxicities of cisplatin (CDDP)- and carboplatin (CBDCA)-based chemotherapy (CT) in patients with SCCHN.

**Methods:**

The search strategy included Pubmed, Science Direct, the Cochrane Library, and the China National Knowledge Internet Web. Statistical analyses were performed using RevMan 5.2. The primary endpoint was overall survival (OS) with secondary endpoints of locoregional control (LRC) and grade≥3 toxicity.

**Results:**

Overall, 12 studies and 1165 patients were included. CDDP-based CT significantly improved 5-year OS (HR=0.67, 95% CI, 0.49 to 0.91; *P*=0.01) compared to the CBDCA group. No difference in the 3-year OS/LRC was observed, but a subgroup analysis showed a better 3-year OS in the CDDP arm for non-nasopharynx carcinoma (non-NPC) SCCHN (HR=0.66, 95% CI, 0.48 to 0.91; *P*=0.01). The CDDP-based CT was associated with more gastrointestinal toxicities (RR=4.58; *P*=0.005) and nephrotoxicity (4/110=3.6%) compared to the CBDCA group, but fewer anemia, leukopenia and thrombocytopenia with RRs of 0.27, 0.71, and 0.28 respectively.

**Conclusions:**

Patients with CDDP-based CT can achieve a higher OS, but there is no significant difference in LRC. The CDDP-based CT is associated with fewer hematological toxicities but more gastrointestinal toxicities and nephrotoxicity compared to the CBDCA arm.

## INTRODUCTION

More than 500,000 people worldwide are diagnosed with squamous cell carcinoma of the head and neck (SCCHN) each year [1, 2]. Several treatment approaches exist for SCCHN, such as radiotherapy, concomitant chemoradiation (CRT), neoadjuvant chemotherapy and adjuvant chemotherapy. In the (MACH-NC) meta-analysis update published in 2011, results from 87 randomized clinical trials with 16,192 patients revealed a clear benefit with the use of chemotherapy in all tumor locations of SCCHN, with a hazard ratio (HR) between 0.87 and 0.88 [3]. The (MAC-NPC) meta-analysis was updated in 2014 and included 19 trials and 4798 patients, which showed that the benefit of the addition of CT was consistent for all endpoints: progression-free survival (HR 0.76 [0.70; 0.82], *P*<0.0001), loco-regional control (HR 0.74 [0.65; 0.85], *P*<0.0001), distant control (0.68 [0.60; 0.76], *P*<0.0001) and NPC related mortality (0.73 [0.66; 0.81], *P*<0.0001). There was a significant interaction between treatment effect on OS and the timing of CT (*P*=0.01) in favor of concomitant CT (without adjuvant CT: HR 0.80 [0.70; 0.93]; with adjuvant CT: HR 0.65 [0.56; 0.76]) compared to induction CT alone (HR 0.96 [0.80; 1.16]) or adjuvant CT alone (HR 0.87 [0.68; 1.12]) [4]. According to the National Comprehensive Cancer Network (NCCN) Guidelines of head and neck cancers, Version 2.2014, adjuvant chemotherapy and induction chemotherapy are revised in the recommendation for category 2A and 3, respectively, while concurrent chemoradiotherapy is now a category 1 recommendation for suggested standard therapy.

The platinum-based (mainly cisplatin and carboplatin) concurrent chemoradiotherapy regimens are recommended by the NCCN Guidelines of head and neck cancers, Version 2.2014, and cisplatin has priority over the other platinum-based drugs. The benefit of combining cisplatin with radiation therapy has been confirmed in multiple randomized clinical trials [5, 6, 7]. However, significant cisplatin-induced toxicities include myelosuppression, nausea and vomiting, nephrotoxicity [8], mucositis, dermatitis, and potentially permanent ototoxicity. In this setting, there is currently uncertainty regarding the best choice of concomitant agent.

Carboplatin, a second generation platinum-based drug, has been frequently used to replace cisplatin because of its similar mode of action, but lower rates of ototoxicity, nephrotoxicity, neurotoxicity and emesis [9]. A randomized controlled trial compared concurrent chemotherapy with carboplatin versus standard concurrent chemoradiotherapy with cisplatin in 206 patients with locoregionally advanced nasopharyngeal caner (NPC). There was no significant difference between the two groups in terms of 3-year overall survival (cisplatin 77.7%, carboplatin 79.2%, HR=0.83, *P*=0.9884) and 3-year disease-free survival (cisplatin 63.4%, carboplatin 60.9%, HR=0.70, *P*=0.9613). The tolerability of the carboplatin-based regimen was better than that of the cisplatin-based regimen [10]. Currently, there have also been several other studies comparing the cisplatin-based regimen with the carboplatin-based regimen [11–21], but none of these studies were sufficiently large to demonstrate a statistically significant effect.

Thus, we performed a meta-analysis of the published clinical trials, retrospective studies and matched-pair analysis comparing the cisplatin-based regimen with the carboplatin-based regimen in SCCHN. Both treatment groups were compared for overall survival (OS) and locoregional control (LRC), as well as for toxicities.

## RESULTS

### Selection of studies

**Figure 1:**
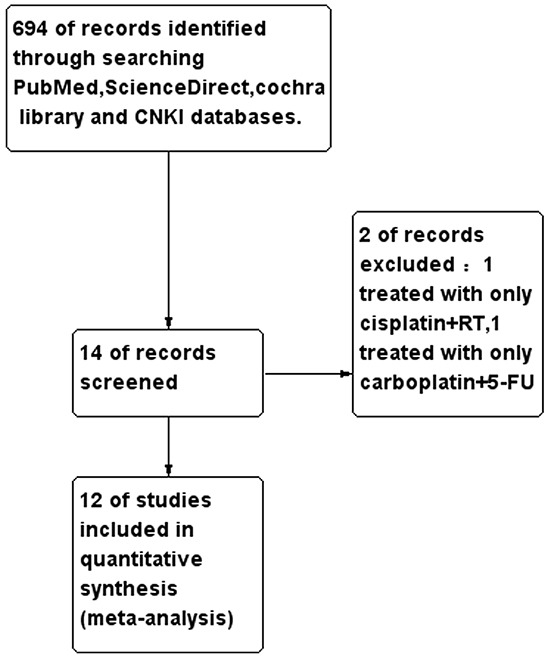
Consort diagram outlining the study selection

A total of 694 studies were retrieved using the search strategies, and 12 studies published between 1995 and 2013 were included in the final analysis (Figure 1). All of the studies included both regimens. Overall, 1165 patients from the selected studies were included in this analysis. The number of patients treated with cisplatin and carboplatin was 593 and 572, respectively. The median age ranged between 46-77 and 50-73 for the cisplatin and carboplatin groups, respectively. Not all of the outcome data were reported or extractable from all of the included studies. Details of the selected studies are provided in Table 1.

**Table 1:** Study characteristics and summary findings

Study	N	Design	Demographics Median age (years)	Gender (M/F)	Patient population	Treatment arms	3year OS(%)	3year LRC(%)	5year OS(%)	5year LRC(%)
**Kua VF et al. 2013**	41	retrospective study	All above 30	CDDP:15/2 CBDCA:15/9	metastatic and recurrent SCCHN and NPC Race: Malays, Chinese and India	CDDP: CDDP 75mg/m^2^,D_1_+5FU 750 mg/m^2^, D_1-5_ (*n* = 17) CBDCA: CBP AUC 5, D_1_+5FU 500 mg/m^2^ D_1-2_+5FU 500mg/m^2^, D_1-2_, bolus (*n* = 24)	- -	- -	- -	- -
**A.C.Wilkins et al.2013**	130	matched-pair analysis	CDDP:58 CBDCA:59	CDDP:51/11 CBDCA:49/16	locally advanced SCCHN(except NPC) Stage III-IV. United Kindom	CDDP: RT+DDP (100mg/m^2^, D_1__,29_ (*n* = 65) CBDCA: RT+CBP (AUC5 D_1,29_ (*n* = 65)	Cis:68 Cb:59	Cis:79 Cb:87	Cis:61.5 Cb:55.4	- -
**M.Kreppel et al.2012**	53	retrospective study	-	-	locally advanced squamous cell carcinoma of maxillary sinus, stage II-IV. Germany	CDDP: Neoadjuvant RCT (RT+DDP 40mg/m^2^/day,D_1-5_)+surgery (*n* = 33) CBDCA: Neoadjuvant RCT (RT+CBP 70mg/m^2^/day)+ surgery (*n* = 20)	- -	- -	Cis:37.2 Cb:31.7	Cis:63.9 Cb:49.4
**D.Rades et al.2012**	106	retrospective study	-	CDDP:47/18 CBDCA:32/9	locally advanced squamous cell carcinoma of oropharynx and oral cavity (stage III/IV) Germany	CDDP: Surgery+ CCRT: RT+DDP20 mg/m^2^,D_1—5_, D_29—33_ (*n* = 65) CBDCA: Surgery+ CCRT :RT+ CBP AUC 1.5,D_1—5_, D_29—33_ (*n* = 41)	Cis:78 Cb:51	Cis:85 Cb:62	Cis:66.6 Cb:37.6	- -
**I.Chitapanarux et al.2007**	206	Randomized,non-inferiority trial	CDDP:46 CBDCA:50	CDDP:57/44 CBDCA:69/36	locally advanced NPC (stage III-IV) Thailand	CDDP:CCRT:RT+DDP(100mg/m^2^/day,D_1,22,43_)+Ad-CT:DDP(80mg/m^2^)+5-FU (1000mg/m^2^/day,96h), every 4 weeks×3 cycles (*n* = 110) CBDCA:CCRT:RT+CBP(100mg/m^2^/day,D_1,8,15,22,29,36_)+Ad-CT:CBP:AUC5+5-FU (1000mg/m^2^/day,96h), every 4 weeks×3 cycles (*n* = 110)	Cis:78.6 Cb:79.8	- -	- -	- -
**A.Homma et al.2004**	119	randomized, phase II study	CDDP:61.5 CBDCA:62	CDDP:54/5 CBDCA:59/1	Locally SCCHN, excluding cancers of glottic region, NPC, and paranasal sinus lesions, stageII-IV Japan	CDDP:CCRT: DDP4mg/m^2^/day,D_1-28_+RT (*n* = 59) CBDCA: CCRT: CBP100mg/m^2^,D_1.8.15.22_ +RT (*n* = 60)	Cis:68.5 Cb:80.2	Cis:38.2 Cb:57.4	Cis:66 Cb:71.4	Cis:35.5 Cb:56.2
**Deng LC et al.1999**	57	retrospective study	CDDP:51 CBDCA:53	CDDP:20/11 CBDCA:18/8	locally advanced SCCHN, stage III-IV. P.R.China	CDDP: Neoadjuvant CT (DDP100 mg/m^2^,D_1_+5FUD_1-5_,1000mg/m^2^)+RT (*n* = 31) CBDCA: Neoadjuvant CT (CBP300 mg/m^2^,D_1_+5FU,D_1-5_,1000mg/m^2^)+RT (*n* = 26)	-	-	-	-
**Deng KK2009**	74	Prospective non-randomized control study	CDDP:55 CBDCA:54	CDDP:25/13 CBDCA:24/12	Stage II-IV NPC P.R.China	CDDP: induction CT (DDP 20mg/m^2^+5-FU 750mg/m^2^,D_1-5_)+CCRT(DDP20mg/m^2^+5-FU750mg/m^2^,D_21-26,43-48_+RT) (*n* = 38) CBDCA: induction CT (CBP50mg/m^2^+5-FU750mg/m^2^,D_1-5_)+CCRT(CBP50mg/m^2^+5-FU750mg/m^2^,D_21-26,43-48_+RT) (*n* = 36)	- -	- -	- -	- -
**Wen QL et al.2013**	176	retrospective study	- -	CDDP:49/39 CBDCA:52/36	Locally advanced NPC, stage III-IV P.R.China	CDDP: CCRT: RT+DDP40mg/m^2^, weekly (*n* = 88) CBDCA: CCRT: RT+CBP60mg/m^2^, weekly (*n* = 88)	- -	- -	- -	- -
**Ge W et al.1998**	34	retrospective study	- -	CDDP:13/4 CBDCA:13/4	Middle and advanced NPC, N2-N3 P.R.China	CDDP: neoadjuvant CT (DDP 100mg/m^2^D_1_+5-FU1000mg/m^2^D_1-5_)+RT (*n* = 17) CBDCA: neoadjuvant CT (CBP 300mg/m^2^D_1_+5-FU 1000 mg/m^2^D_1-5_)+RT(*n* = 17)	- -	- -	- -	- -
**De Andres et al.1995**	95	Prospective,randomized,nonblind trial	CDDP:55 CBDCA:50	CDDP:46/3 CBDCA:43/3	Locally advanced SCCHN, stage IV-M0 except NPC Spain	CDDP: neoadjuvant CT (DDP 100mg/m^2^+5-FU5000mg/m^2^,120h,,D_1,22,43_)×3 courses+RT (*n* = 49) CBDCA: neoadjuvant CT (CBP 400mg/m^2^,24h+5-FU5000mg/m^2^,120h,D_1.22.43_)×3 courses+RT (*n* = 46)	- -	- -	Cis:49 Cb:25	- -
**N Fuwa et al.2008**	60	retrospective study	CDDP:77 CBDCA:73	-	Locally advanced oral cavity cancer, stage III-IV, except carcinoma of the base of tongue Japan	CDDP: CCRT:RT+DDP (continuous arterial injection,20-30mg/m^2^,repeated 6-7 times) (*n* = 21) CBDCA: CCRT:RT+CBP(arterial injection, AUC 6-8,repeated 6-7 times) (*n* = 39)	Cis:55.07 Cb:30.77	Cis:61.39 Cb:60.58	-	-

DDP/ Cis= cisplatin; CBP/ Cb= carboplatin.

### Overall survival

**Figure 2:**
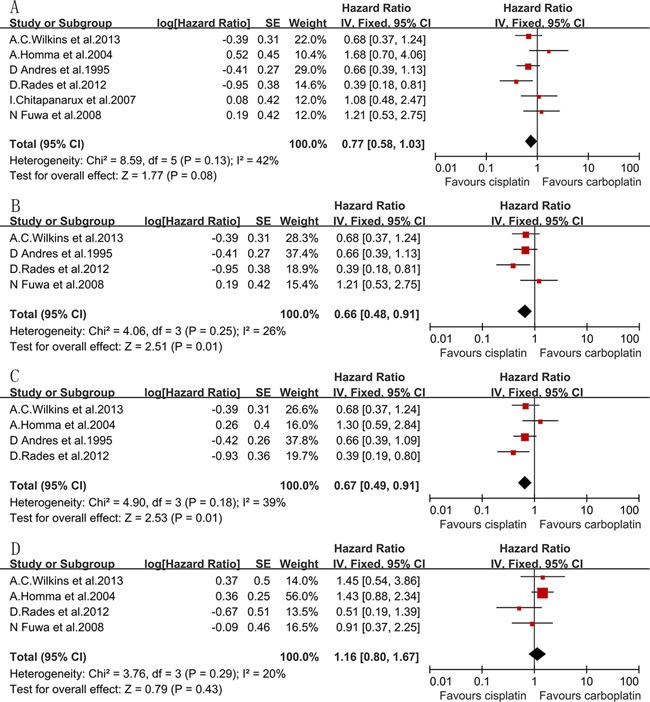
Forest plots of the hazard ratio of A 3-year OS, B. 3-year OS for non-NPC SCCHN, C. 5-year OS, D. 3-year LRC

Six studies [10–14, 20] reported the data of 3-year OS, which included 369 patients in the cisplatin group and 361 patients in the carboplatin group. The 3-year OS for the cisplatin group was statistically similar to that of the carboplatin group (HR=0.77, 95%CI, 0.58 to 1.03; *P*=0.08) (Figure 2A). Interestingly, the total dose of cisplatin in one trial [12] (112 mg/m^2^) was much lower than those used in other studies reported in the literature, and in the subgroup analysis of non-NPC SCCHN, the heterogeneity was raised to a critical value (I^2^=49%, *P*=0.10) if this trial was included. After the exclusion of this trial, the cisplatin-based chemotherapy improved 3-year OS compared with the carboplatin-based chemotherapy, with an HR of death of 0.66 (95% CI, 0.48 to 0.91; *P*=0.01) (Figure 2B). All of the included studies were treated with concurrent chemoradiotherapy (CCRT), except one trial [14], which used neoadjuvant CT+RT. Without this trial, the 3-year OS was not significantly different between the two groups (HR=0.73, 95% CI, 0.50 to 1.05; *P*=0.09). There was no heterogeneity between studies for the 3-year OS analyses.

Four eligible studies [11–14] had data for 5-year OS, including 238 patients in the cisplatin group and 212 patients in the carboplatin group. The HR of 5-year OS was 0.67 (95% CI, 0.49 to 0.92; *P*=0.01) (Figure 2C), which showed a significant difference in favor of the cisplatin group. All of the four included studies were performed in non-NPC SCCHN patients. Among the four included studies, only one study [14] treated with neoadjuvant CT+RT, and the other studies treated with concurrent RCT. In the subgroup analysis of 5-year OS for CCRT-treated patients, we removed only one study [12] due to the heterogeneity, which was related to its lower dose of cisplatin (I^2^=59%, *P*=0.09), and with an HR of 0.54 (95% CI, 0.34 to 0.85; *P*=0.008), it also showed a significantly higher rate of 5-year OS in favor of cisplatin. There was no heterogeneity between studies for the 5-year OS analyses.

### Locoregional control

Four studies [11–13, 20] were included in the 3-year LRC analysis, including 210 patients in the cisplatin group and 205 patients in the carboplatin group. There was no significant difference between the two arms for the 3-year LRC (HR=1.16, 95% CI, 0.80 to 1.67; *P*=0.43) (Figure 2D). No heterogeneity was observed. The CCRT was planned in these four studies, which were all designed for non-NPC SCCHN patients.

### Toxicities

#### Grade≥3 nausea and vomiting

**Figure 3:**
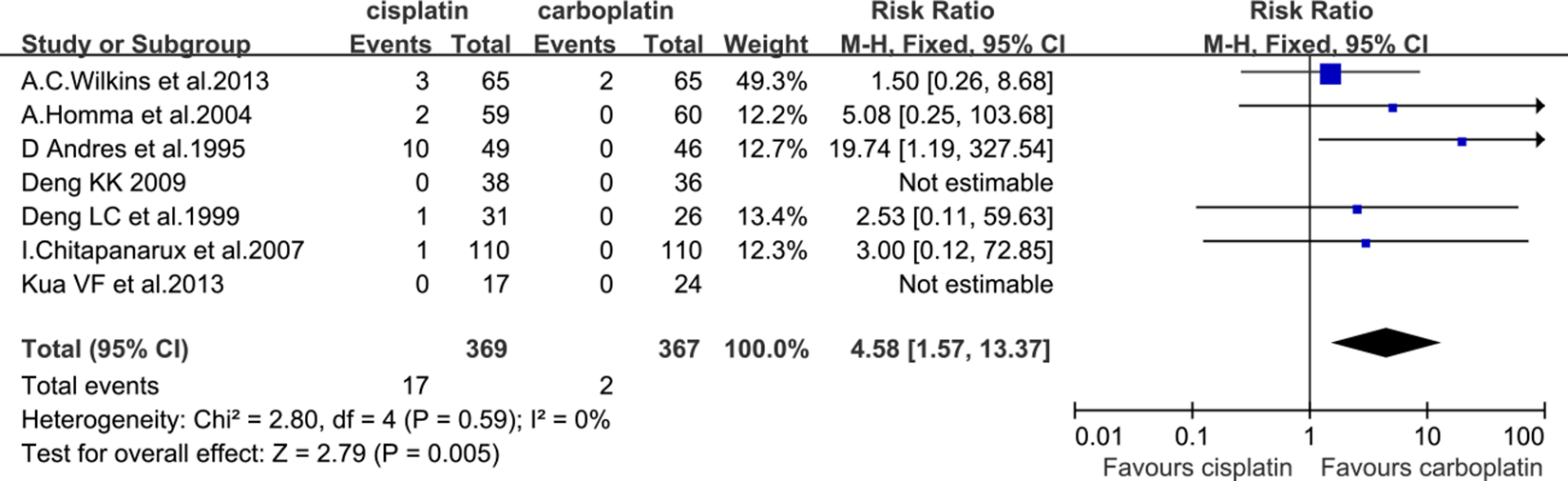
Forest plots of the risk ratio of grade≥3 nausea and vomiting

Seven eligible studies [10–12, 14–16, 18] had the data for grade≥3 nausea and vomiting, which included 369 patients in the cisplatin group and 367 patients in the carboplatin group. There was a significant difference in favor of the carboplatin group (RR=4.58, 95% CI, 1.57 to 13.37; *P*=0.005) (Figure 3). All of the included studies were treated with concurrent CRT, except two studies [14, 16], which were treated with neoadjuvant CT+RT. After rejecting these two studies, the carboplatin group was also associated with a lower rate of grade≥3 nausea and vomiting, with an RR of 2.34 (95% CI, 0.62 to 8.91; *P*=0.21), but the difference did not reach statistical significance. There was also no heterogeneity between studies for grade≥3 nausea and vomiting, independent of whether the included studies were treated with concurrent CRT only. Most of the participants in the three studies [10, 15, 16] were diagnosed with NPC, and the risk ratio of grade≥3 nausea and vomiting in NPC was 2.76 (95% CI, 0.29 to 25.96; *P*=0.38; heterogeneity *P*=0.94, I^2^=0%). In the other three studies, which excluded NPC patients, the incidence of grade≥3 nausea and vomiting was found to be lower in the carboplatin group (RR=5.21; 95% CI, 1.53 to 17.79; *P*=0.008; heterogeneity *P*=0.25, I^2^=28%).

#### Grade≥3 mucositis

**Figure 4:**
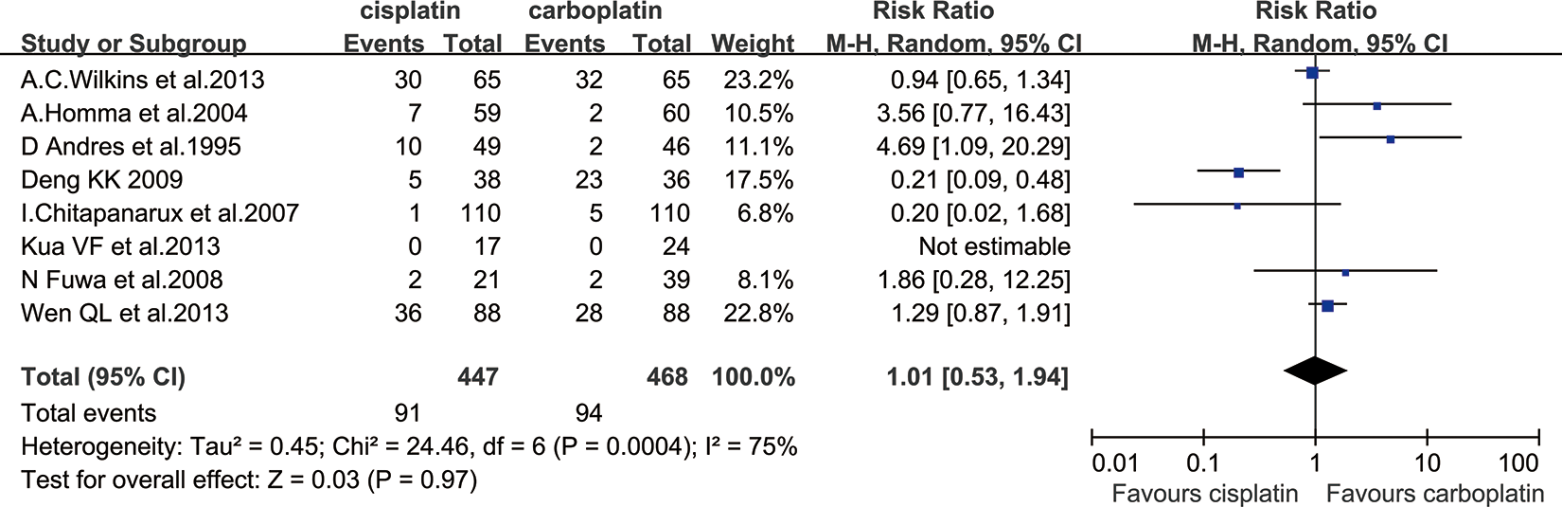
Forest plots of the risk ratio of grade≥3 mucositis

Eight eligible studies [10–12, 14, 15, 18, 20, 21] had the data for grade≥3 mucositis, which included 447 patients in the cisplatin group and 468 patients in the carboplatin group. No difference in grade≥3 mucositis was observed (RR=1.01; 95% CI, 0.53 to 1.94; *P*=0.97). A between trial heterogeneity was observed for grade≥3 mucositis with an I^2^=75% (*P*=0.0004) (Figure 4). For the six studies treated with concurrent CRT [10–12, 15, 20, 21], there was also a nonsignificant difference between the two groups (RR=0.84, 95%CI, 0.43 to 1.62; *P*=0.60), while significant heterogeneity existed among studies (I^2^=75%, *P*=0.001). There was no significant difference between the two groups for NPC patients [10, 15, 18, 21]] (RR=0.43; 95% CI, 0.09 to 2.03; *P*=0.28), but there was significant heterogeneity with an I^2^=89%. According to the results of the sensitivity analysis, one trial [21] was excluded, and there was a significant difference in favor of the cisplatin group (RR=0.20; 95% CI, 0.09 to 0.45; *P*<0.0001). For the non-NPC patients [11, 12, 14, 20]], the rate of grade≥3 mucositis was not found to be significantly different between the two groups (RR=1.99; 95% CI, 0.73 to 5.41; *P*=0.18), but the heterogeneity was also significant with an I^2^=63% (*P*=0.04). According to the results of the sensitivity analysis, one trial [11] was excluded, and the carboplatin group was associated with a lower rate of grade≥3 mucositis, with an RR of 3.55 (95% CI, 1.42 to 8.88; *P*=0.007).

#### Grade≥3 skin toxicity

Five eligible studies [10–12, 15, 21] reported the data for grade≥3 skin toxicity, which included 360 patients in the cisplatin group and 359 in the carboplatin group. There was no significant difference in grade≥3 skin toxicity between the two groups (RR=1.06; 95% CI, 0.74 to 1.51; *P*=0.75; heterogeneity *P*=0.31, I^2^=16%). The five studies were all treated with CCRT. Most participants in the three studies [10, 15, 21] had NPC, and there was no significant difference in grade≥3 skin toxicity between the two groups in NPC (RR=0.99; 95% CI, 0.66 to 1.50; *P*=0.98). No heterogeneity was observed. The other two studies [11, 12] excluded patients with NPC and showed no difference in grade≥3 skin toxicity for non-NPC SCCHN (RR=1.47; 95% CI, 0.34 to 6.29; *P*=0.60), and there was significant heterogeneity with an I^2^ equivalent to 65%.

#### Grade≥3 anemia

**Figure 5:**
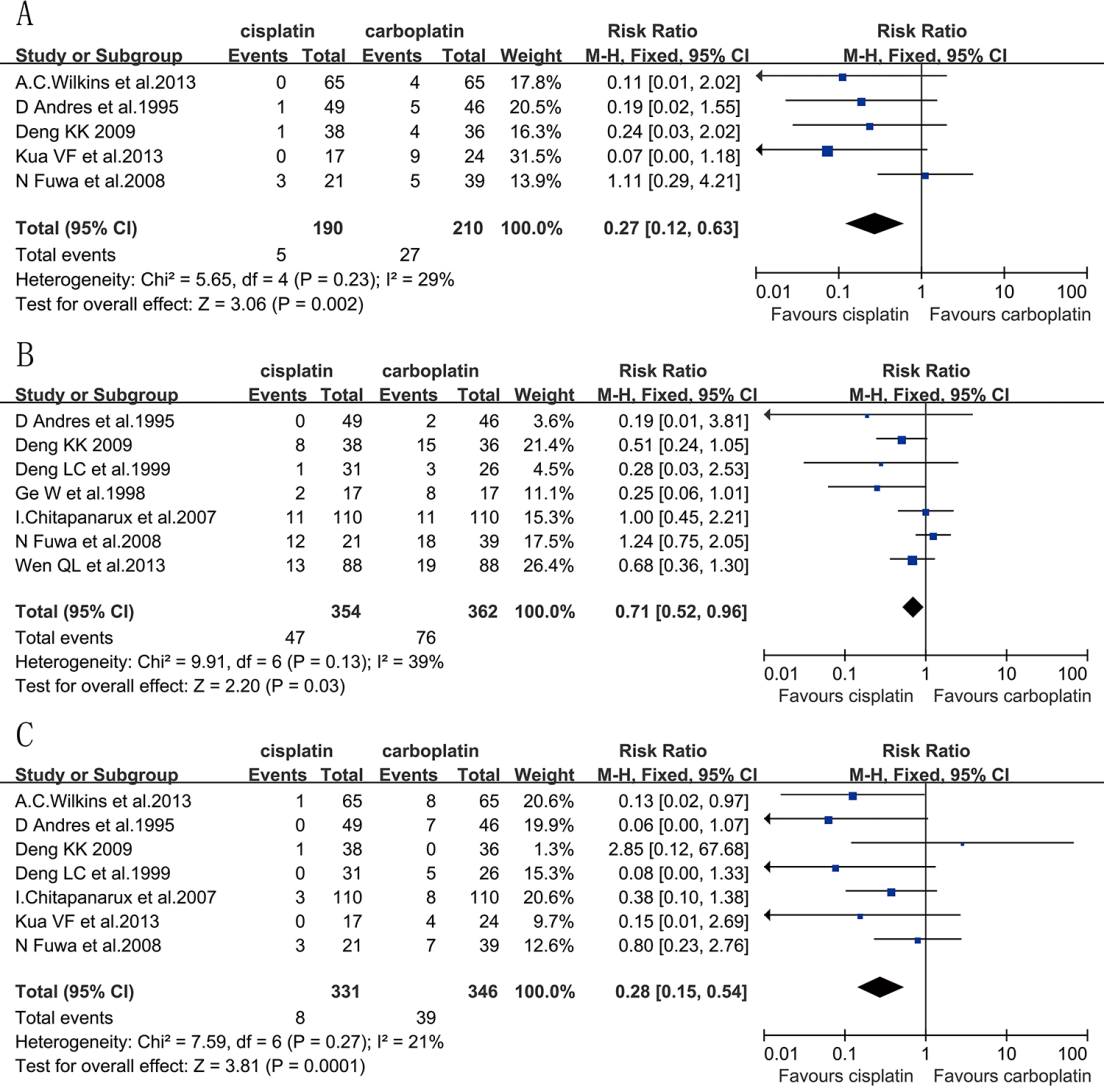
Forest plots of the risk ratio of grade≥3 hematologic toxicities, A. anemia, B. leukopenia, C. thrombocytopenia

Six eligible studies [10, 11, 14, 18, 20] reported the data for grade≥3 anemia, which included 300 patients in the cisplatin group and 320 patients in the carboplatin group. The RR was 0.48 (95% CI, 0.11 to 2.11; *P*=0.33), showing a nonsignificant difference of grade≥3 anemia between the two groups. However, significant heterogeneity existed among trials (*P*=0.006, I^2^=70%). This heterogeneity was correlated to one study [10] due to the higher proportion of female participants than the other included studies. After removing this study, there was a significant difference in grade≥3 anemia in favor of the cisplatin group (RR=0.27; 95% CI, 0.12 to 0.63; *P*=0.002; heterogeneity *P*=0.23, I^2^=29%) (Figure 5A). To analyze the grade≥3 anemia of the studies that were treated with only concurrent CRT, two studies [14, 18] were excluded, and there was no significant difference between the two groups (RR=0.44; 95% CI, 0.17 to 1.17; *P*=0.10; heterogeneity *P*=0.22, I^2^=34%). Only one study [15] was limited to NPC patients. Three studies [11, 14, 20] were limited to SCCHN excluding NPC. Cisplatin-based chemotherapy was also associated with a non-significant lower rate of grade≥3 anemia for non-NPC SCCHN compared with carboplatin, with an RR of 0.41 (95% CI, 0.16 to 1.07; *P*=0.07). No heterogeneity was observed.

#### Grade≥3 leukopenia

Seven eligible studies [10, 14–17, 20, 21] had data for grade≥3 leukopenia, which included 354 patients in the cisplatin group and 362 patients in the carboplatin group. Cisplatin-based regimen improved grade≥3 leukopenia as compared with a regimen based on carboplatin, with an RR of 0.71 (95% CI, 0.52 to 0.96; *P*=0.03; heterogeneity *P*=0.13, I^2^=39%) (Figure 5B). Three studies [14, 16, 17] were excluded because their treatment regimen was neoadjuvant CT+RT, whereas the remaining four studies [10, 15, 20, 21]] were treated with concurrent CRT. Grade≥3 leukopenia was estimated to be statistically similar between the two arms (RR=0.82; 95% CI, 0.59 to 1.13; *P*=0.22; heterogeneity *P*=0.18, I^2^=38%). Five studies [10, 15–17, 21] were largely limited to NPC patients, and the grade≥3 leukopenia occurrence rate was significantly lower in the cisplatin arm (RR=0.61; 95% CI, 0.42 to 0.90; *P*=0.01; heterogeneity *P*=0.42, I^2^=0%). In studies for non-NPC SCCHN patients [14, 20], grade≥3 leukopenia was statistically similar between the two groups (*P*=0.82). There was no heterogeneity observed.

#### Grade≥3 thrombocytopenia

Seven eligible studies [10, 11, 14–16, 18, 20] reported the data of grade≥3 thrombocytopenia, which included 331 patients in the cisplatin group and 346 in the carboplatin group. There was a significant difference in grade≥3 thrombocytopenia in favor of the cisplatin group (RR=0.28; 95% CI, 0.15 to 0.54; *P*=0.0001) (Figure 5C). Among the seven included studies, two [14, 16] were treated with neoadjuvant CT+RT, one [18] was treated with palliative CT, and the rest of the studies were treated with concurrent CRT. Three non-concurrent CRT studies were excluded [14, 16, 18], and the risk ratio of grade≥3 thrombocytopenia was 0.44 (95% CI, 0.21 to 0.92; *P*=0.03), which also showed a significant reduction in the risk of grade≥3 thrombocytopenia in favor of the cisplatin arm. There was no heterogeneity between trials for grade≥3 thrombocytopenia analysis. Most of the participants in the three studies [10, 15, 16] were diagnosed with NPC, and there was also a significant difference in favor of the cisplatin group for NPC (RR=0.34; 95% CI, 0.13 to 0.92; *P*=0.03; heterogeneity *P*=0.25, I^2^=29%). The other three studies [11, 14, 20] regarded NPC as an exclusion criteria, and showed a significantly lower rate of grade≥3 thrombocytopenia in the cisplatin group (RR=0.26; 95% CI, 0.10 to 0.65; *P*=0.004). However, significant heterogeneity existed among studies (*P*=0.10, I^2^=56%). According to the results of a sensitivity analysis, one trial [20] was excluded, and there was a significant difference in grade≥3 thrombocytopenia in favor of the cisplatin group (RR=0.09; 95% CI, 0.02 to 0.49; *P*=0.005; heterogeneity *P*=0.70, I^2^=0%).

Overall, mucositis and leukopenia were the most frequent disorders observed in patients in both groups. There were 3 treatment-related deaths in the cisplatin group and 5 in the carboplatin group of the two studies [12, 18]. In addition to the adverse events described above, there were also some other events reported, such as severe ototoxicity, neurotoxicity, dysphagia, hepatotoxicity and other symptoms. Grade≥3 nephrotoxicity only appeared in one study [10], whereas overall, the five studies [10, 11, 15, 18, 20] had data for this toxicity. For the cisplatin group, four patients suffered grade 3-4 nephrotoxicity. However, no patients experienced this severe toxicity in the carboplatin group.

## DISCUSSION

To the best of our knowledge, this article is the first meta-analysis to evaluate the efficacy and toxicity of cisplatin-based CT versus that based on carboplatin for moderate to advanced SCCHN. A total of 1165 patients from 12 studies, with 593 patients in the cisplatin group and 572 patients in the carboplatin group, were analyzed. Cisplatin significantly improved the 5-year OS and severe hematological toxicities (grade≥3 anemia, leukopenia and thrombocytopenia) compared with carboplatin for SCCHN. In contrast, carboplatin was associated with a lower rate of gastrointestinal toxicities (grade≥3 nausea and vomiting) and nephrotoxicity. No significant difference between the two arms was observed in the 3-year LRC and severe skin toxicity.

CCRT was planned in eight studies (924 patients) [10–13, 15, 19–21]. In the SCCHN patients treated with CCRT, cisplatin also showed a marked improvement in the 5-year OS and rate of grade≥3 thrombocytopenia compared with carboplatin. There was no significant difference in the 3-year OS, 3-year LRC and other toxicities between the carboplatin and cisplatin cohorts for concurrent CRT-treated SCCHN.

Four studies [10, 15, 17, 21]] fulfilled the inclusion criteria of all participants with nasopharyngeal carcinoma (NPC), and the participants of two studies [16, 18] were mostly diagnosed with NPC (73.68% and 65.85%). There was a remarkable difference in favor of cisplatin in severe hematotoxicity (grade≥3 anemia, leukopenia and thrombocytopenia) for NPC. Six studies [11–14, 19, 20] were designed for non-NPC SCCHN patients. For non-NPC SCCHN patients, the 3-year and 5-year OS were significantly higher in the cisplatin arm, while carboplatin-based chemotherapy significantly improved severe gastrointestinal toxicity (grade≥3 nausea and vomiting). There was more grade≥3 thrombocytopenia in the carboplatin arm.

Mucositis is one of the most serious problems observed in patients treated for head and neck squamous-cell cancer. This side effect is observed in more than 80% of RT-treated patients and can last for more than 5 weeks [22]. Mucositis causes substantial pain, bleeding, interferes with the patient's ability to eat, and worsens the patient's quality of life. In some cases, the severe mucosal toxicity and associated discomfort can even result in incomplete radiation doses and poor local tumor control, which may adversely affect survival [23]. Our subgroup analysis of mucositis appears to favor carboplatin-based regimens in non-NPC patients, and the result is reversed in NPC patients. After confirming the data of the four studies [10, 15, 18, 21]] included for the analysis of mucositis in NPC patients, we found that in one Chinese study [15], the dosage and frequency of carboplatin were much higher than those recommended in the instructions for Asian people. In this study, the carboplatin group underwent chemotherapy using carboplatin 50 mg/m^2^ for d1-5, 5-FU 75-mg/m^2^ for d1-5, repeated every 3 weeks for 3 cycles. According to the instructions of carboplatin for Asian patients, regular dosages of carboplatin are 200-400 mg/m^2^, d1, repeated every 3-4 weeks or 50 mg/m^2^, d1-5, repeated every 4 weeks. The severe toxicities and poor ability to receive all of the carboplatin courses may attribute to the higher dosage and frequency for carboplatin. This may be a potential risk factor affecting mucositis analysis, which motivates additional efforts for the standardization of a definitive setting in large randomized trials to indicate superiority of these two regimens in the subgroup analysis.

Most of the studies reporting the data of 3/ 5-year OS and 3-year LRC were of concurrent radiochemotherapy for non-NPC SCCHN patients, and thus, we can conclude that cisplatin appears preferable to carboplatin for the concurrent radiochemotherapy of patients with non-NPC SCCHN because it results in better 3 / 5-year OS. There was no significant difference in 3-year LRC. Severe hematological toxicities (grade≥3 anemia, thrombocytopenia and leukopenia) were more frequent after carboplatin treatment, particularly for NPC. Severe gastrointestinal toxicity (grade≥3 nausea and vomiting) was more frequent in the cisplatin group, particularly for non-NPC SCCHN patients.

A major limitation of this meta-analysis is that there are only three randomized trials available, while others are retrospective studies or matched-pair studies. The second limitation is that the studies reporting the OS and LRC were mostly performed in non-NPC SCCHN patients using concurrent radiochemotherapy, and the data of OS and LRC in six studies [15–19, 21] are missing. Third, the treatment models of concurrent radiochemotherapy varied from study to study, including chemotherapy administered every week, every day, every 3 weeks or the first week. This variation may affect the results of the analysis. Finally, the data of late toxicity, such as hearing loss, xerostomia and radiation encephalopathy are missing. We analyzed the acute toxicities only in the present study.

In conclusion, our research indicated that compared with the carboplatin group, cisplatin-based CT in non-NPC SCCHN patients could improve the 3-year OS and 5-year OS, while the 3-year LRC was not significantly different between the two groups. Severe hematologic toxicities (grade≥3 anemia, leukopenia and thrombocytopenia) were more frequent in the carboplatin group, particularly for NPC patients. There was more severe gastrointestinal toxicity (grade≥3 nausea and vomiting) in the cisplatin group, particularly for non-NPC SCCHN patients. Larger and multicenter RCTs are required to assess whether the cisplatin-based regimen is superior to that based on carboplatin for various types of moderate to advanced SCCHN. Moreover, RCTs comparing different regimens of concurrent radiochemotherapy, such as chemotherapy administered every week, every day, every 3 weeks or the first week should also be explored in moderate to advanced SCCHN.

## MATERIALS AND METHODS

### Search strategy

The literature search was performed using PubMed, Science Direct, the Cochrane library and the CNKI databases on abstracts published from 1983 to 2014 using keywords [head and neck neoplasms OR head and neck cancer OR head and neck tumor OR head-neck tumors] AND cisplatin AND carboplatin. We also retrieved “lip,” “oral cavity,” “oropharyngeal,” “hypopharyngeal,” “nasopharyngeal,” “laryngeal,” “sinus,” and “salivary gland.” Three randomized clinical trials, eight retrospective studies and one matched-pair analysis fulfilled the search criteria of therapy with cisplatin versus carboplatin in a randomized clinical trial, retrospective study or matched-pair analysis.

### Data collection and analysis

The extracted data included demographics, treatment and clinical outcomes [overall survival (OS), loco-regional control (LRC) and toxicities]. The outcome data extracted for each arm were analyzed using random and fixed effect models and were reported as weighted measures. All analyses were performed using RevMan 5.2 software (Cochrane Collaboration's Information Management System). The risk ratios of the adverse effect were calculated, which were presented with the corresponding 95% confidence interval (CI). The results of OS and LRC were also extracted. The significant outcome was defined as a *P*-value below 0.05; a fixed-effects model was applied when homogeneity was good (P≥0.10, I^2^≤50%), or a random-effects model was used. Kaplan-Meier curves were evaluated using the Engauge-Digitizer.
